# *Listeria monocytogenes* in Fresh Produce: Outbreaks, Prevalence and Contamination Levels

**DOI:** 10.3390/foods6030021

**Published:** 2017-03-09

**Authors:** Qi Zhu, Ravi Gooneratne, Malik Altaf Hussain

**Affiliations:** Department of Wine, Food and Molecular Biosciences, Lincoln University, Lincoln 7647, Canterbury, New Zealand; Qi.Zhu@lincolnuni.ac.nz (Q.Z.); Ravi.Gooneratne@lincoln.ac.nz (R.G.)

**Keywords:** *Listeria monocytogenes*, fresh produce, foodborne pathogen, contamination, listeriosis

## Abstract

*Listeria monocytogenes*, a member of the genus *Listeria*, is widely distributed in agricultural environments, such as soil, manure and water. This organism is a recognized foodborne pathogenic bacterium that causes many diseases, from mild gastroenteritis to severe blood and/or central nervous system infections, as well as abortion in pregnant women. Generally, processed ready-to-eat and cold-stored meat and dairy products are considered high-risk foods for *L. monocytogenes* infections that cause human illness (listeriosis). However, recently, several listeriosis outbreaks have been linked to fresh produce contamination around the world. Additionally, many studies have detected *L. monocytogenes* in fresh produce samples and even in some minimally processed vegetables. Thus *L. monocytogenes* may contaminate fresh produce if present in the growing environment (soil and water). Prevention of biofilm formation is an important control measure to reduce the prevalence and survival of *L. monocytogenes* in growing environments and on fresh produce. This article specifically focuses on fresh produce–associated listeriosis outbreaks, prevalence in growing environments, contamination levels of fresh produce, and associated fresh produce safety challenges.

## 1. Introduction

*Listeria monocytogenes*, a member of the genus *Listeria*, naturally occurs in agricultural environments such as soil, manure and water [1]. Scientific literature frequently discusses the ability of this microorganism to survive in the food-processing and produce-packing environment and equipment [2]. It is a pathogenic bacterium that can cause a rare but dangerous infection called listeriosis. The severity of listeriosis can range from mild gastroenteritis to severe disease conditions (septicemia, encephalitis, meningitis, abortions and stillbirths) and can result in a high fatality rate in immune-compromised populations [3]. Some people have a higher risk for developing listeriosis, such as the elderly (>65 years) [4,5,6], infants and toddlers (<5 years) [4,7,8,9], pregnant women [3,8], and the unborn [8,10]. About 17% of listeriosis cases occur in pregnant women [10]. According to the FDA (Food and Drug Administration), about 2500 people suffer from listeriosis in the USA annually [10]. The mortality rate could be 20%–30% of those who contract listeriosis [3]. *L. monocytogenes* is responsible for 19% of the total deaths due to the consumption of contaminated food in the USA [11].

*L. monocytogenes* is commonly found and isolated from processed, ready-to-eat (RTE) and cold-stored meat and dairy products. An increasing number of recent reports show contamination and prevalence of *L. monocytogenes* in fresh produce. *L. monocytogenes* bacteria have been isolated from market or restaurant produce such as cabbage [12], corn [13], carrots [14,15,16], lettuce [17,18,19,20,21,22,23], cucumbers [1,24,25], parsley [11,26,27] and salad vegetables [11,25,28,29]. Outbreaks of *L. monocytogenes* infections associated with fresh produce have been reported in various parts of the world [24]. For example, *L. monocytogenes* was responsible for the deaths of 10 people in a food poisoning listeriosis outbreak in chopped celery in Texas in 2010 [30]; in 2011, 30 people were infected by listeria-contaminated melons in Colorado [31]; and in 2014, a listeria outbreak linked to caramel apple contamination was reported in California [5]. This trend has continued and prevention of *Listeria* contamination in fresh fruit and vegetables as well as fresh produce–associated listeriosis outbreaks is now a food safety challenge.

This article focuses on fresh produce–associated listeriosis outbreaks, prevalence and survival of *L. monocytogenes* in fresh produce growing environments, listeria contamination of produce and a brief note on measures that could be used to control or reduce the level of contamination.

## 2. Foodborne Listeriosis

It has been over 90 years since human and animal listeriosis was first recognized as an infection caused by a bacterium in the 1920s. The first conclusive link of *L. monocytogenes* to a foodborne outbreak in 1981 stimulated research and survey work to determine the ubiquity of the organism and its method of transmission [32]. *L. monocytogenes* gained recognition as a major foodborne pathogen when the mortality rate did not diminish over the following years, even though the number of cases seemed to be small compared with the estimated illnesses associated with salmonellosis and campylobacteriosis [33].

*L. monocytogenes* can cause two types of disease syndromes. Listeriosis is defined as being caused by invasive *L. monocytogenes*. That is, the organism usually infects sterile parts of the body, such as the liver [34], spleen [35], cerebral spinal fluid [36] and blood [37]. In healthy adults, diarrhea and fever are the main symptoms [38], in pregnant women it is fever, diarrhea, abortion or stillbirth [39], and in the newborn it can cause sepsis, pneumonia and meningitis [40,41,42,43,44]. *L. monocytogenes* can also cause a non-invasive disease, usually as a febrile gastroenteritis or non-invasive gastroenteritis, and it has been linked to outbreaks resulting from contaminated deli meat [45,46], chocolate milk [47], cheese [48,49,50], smoked fish [51,52] and corn [13].

Foodborne listeriosis is a relatively rare but serious disease with a high fatality rate (up to 30%) compared with diseases caused by other foodborne microbial pathogens [2,11]. The incidence rate of foodborne outbreaks caused by contaminated fresh fruits and vegetables has shown an increasing trend in recent years [53]. Most outbreaks have been reported in the USA, Europe, Canada, and to a lesser extent in Australia and New Zealand [33].

## 3. Fresh Produce—Associated Listeriosis Outbreaks

In 1997, a serious *Listeria* outbreak associated with canned corn contaminated by *L. monocytogenes* occurred in two primary schools and a university in Italy. The main symptoms in this outbreak were febrile illness and gastroenteritis. A large number of people (2930 in total) developed febrile gastroenteritis in these three institutes, including primary school students aged six to 10 years, adult staff in the primary school, and students at the university. Investigation into this incident showed that the symptoms occurred after eating food supplied by the same caterer. No other cases were reported outside these three institutions in the same area during 1997 [13].

In 2010, the Texas Department of State Health Services (DSHS) reported a listeriosis outbreak linked to chopped celery. Of the 10 infected patients aged 56 to 93 years admitted to the hospital, five patients died within three months [30].

In 2011, an outbreak of listeriosis occurred in 28 different states in the US, caused by consumption of contaminated melons, in which a total of 147 persons were affected and 33 died. In this outbreak, an analysis of *L. monocytogenes* using pulsed-field gel electrophoresis (PFGE) matched the subtype of *L. monocytogenes* colonies isolated from samples of cut cantaloupe and from the patients’ blood. A pregnant woman who was affected in this outbreak had a miscarriage [54]. In the same year, another outbreak associated with romaine lettuce was recorded across 19 states in the USA. In this outbreak, 84 became sick and of these, 15 died. The Federal Drug Agency (FDA) tested samples randomly from the True Leaf Farms of California. The results of microbiological analyses were positive for *L. monocytogenes*. Approximately 30,000 pounds of chopped and bagged romaine lettuce in 90 cartons were recalled [55].

A listeriosis outbreak associated with caramel apples occurred in December 2014 in the USA. The vehicle for this outbreak was pre-packaged caramel apples. Testing confirmed that the origin of this outbreak was from the firm’s apple-packing facility. In total, 35 people, including 11 pregnant women, were infected by *L. monocytogenes* in 12 states. One of the infected pregnant women had a miscarriage. Seven out of the 35 people infected died during the outbreak [5]. More recently, a multistate outbreak of *L. monocytogenes* affected nine states in the USA in January 2016. Nineteen of the infected people were hospitalized, and one person from Michigan died of listeriosis. Epidemiological and laboratory evidence showed that packaged salads produced in Ohio were responsible for the outbreak. Table 1 below gives a summary of several outbreaks caused by fresh fruit and vegetables since 1979.

## 4. Prevalence and Survival of *L. monocytogenes* in Produce Growing Environments

**Figure 1:**
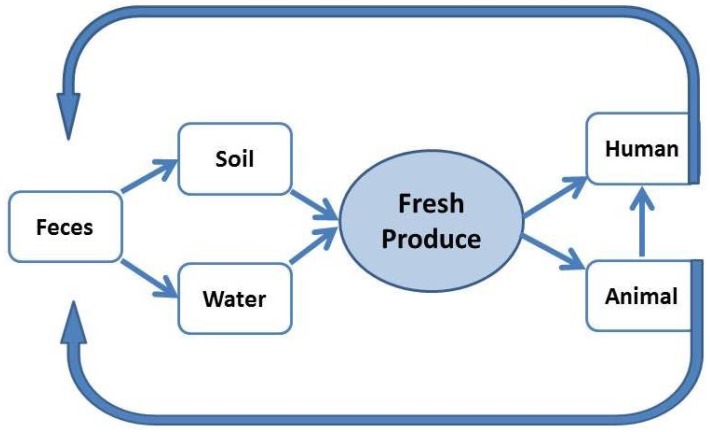
Potential pathways of *L. monocytogenes* transmission to humans via fresh produce.

*L. monocytogenes* is present in many animals and humans [59,60], so it is possible to isolate the bacterium from the feces of these sources and in their environment [61,62,63]. Moreover, fresh produce and soil can be contaminated by sewage water applied as fertilizer to the crop plants [64]. Thus, *L. monocytogenes* can be recycled among vegetables, humans and soils contaminated with feces (Figure 1). This bacterium has an interesting life cycle adaptation capability. It lives a saprophytic life in the soil but can make the transition into a pathogenic life when it enters into human or animal cells [65]. The transition from a saprophyte to a cytosolic pathogen occurs through careful modulation of the activity of a specific regulatory protein (PrfA) and the type of available carbon source.

*L. monocytogenes* has been isolated from RTE foods, such as freshly cut fruit [66] and fresh-cut vegetables [67]. Additionally, *L. monocytogenes* has been isolated from the vegetable growing environment [63]. Temperature, water activity (a_w_) and the pH of foods are the main factors that influence the multiplication and survival of *L. monocytogenes*. Technical reports describe that *L. monocytogenes* can grow under a wide range of growth conditions during food processing and storage, for example, at temperatures as low as −0.4 °C [68] and over a wide range of pH values from 4.3 to 9.4 [69]. In the case of the contaminated melons from Jensen Farms in Colorado (2011), the temperature created an ideal environment for *Listeria* to grow. In addition, the equipment and machinery were impossible to fully clean, and therefore had dirt on them. In addition, the potato washing machine was used for washing cantaloupes. This resulted in the contamination of the cantaloupes. Furthermore, trucks, including those used to haul rejected cantaloupes sent to cattle feedlots, were parked next to the packing plant. This made it easy for the trucks to be contaminated with *Listeria* from the cattle farms [54].

As mentioned above, many factors influence the prevalence of *L. monocytogenes* in fresh produce, including direct or indirect contamination from the environment, such as from soil, water, compost and feces (Table 2). In one research project, 174 samples were tested for *L. monocytogenes* and 48 produced a positive reaction. All *L. monocytogenes*–positive water samples were from natural water sources such as creek and pond water, and none of the 28 samples from piped water and well water tested positive for *L. monocytogenes* [70]. A similar scenario was observed in an investigation into compost and irrigated water [71]. Szymczak et al. [72] conducted research on the prevalence of *L. monocytogenes* in fresh produce in relation to the type of soil, including those lands that were treated with natural fertilizers, artificial fertilizers, and also wastelands and garden plots. It was apparent that the artificial environment was more suitable for *L. monocytogenes* to survive. Exciting research on the factors (including temperature and moisture) that can influence the survival of *L. monocytogenes* in soil was carried out by McLaughlin et al. [73]. They used three marked colonies to monitor *L. monocytogenes* survival in different soil types. They found that *L. monocytogenes* can survive in normal soil, and that the bacterium preferred high-moisture-containing soils. In another research study, Locatelli et al. [74] showed that physical and chemical properties of soil influence the survival of *L. monocytogenes*. Both biotic and abiotic factors influence the survival of *L. monocytogenes*. So, it is quite clear that the external environment (contaminated soil, water and nutrient content, soil properties) affects the survival of *L. monocytogenes*. However, there could be other factors acting concurrently on *L. monocytogenes* survival, especially under moist conditions.

## 5. *L. monocytogenes* Contamination Level of Fresh Produce

Contamination of fresh produce with pathogenic organisms affecting human health can occur at the pre-harvest or post-harvest stage. There are numerous direct or indirect sources of contamination, including animals or insects, soil, water, dirty equipment, and human handling. Many methods, such as the application of antimicrobial agents and UV radiation, have been used to reduce the microbial load in fresh produce. However, a pathogenic bacterium such as *L. monocytogenes* might not be completely inactivated due to its remarkable ability to survive in adverse conditions. In Table 3, several studies are listed that illustrate the prevalence of *L. monocytogenes* in fresh produce. Szymczak et al. [72] showed that 5% of parsley grown in naturally fertilized soil was positive for *L. monocytogenes*. In addition, an assessment of lettuce for *L. monocytogenes* was undertaken from the farm to the table [18]. Results indicated that 1.05 log cfu/g *L. monocytogenes* were found in samples from restaurants and 0.146 log cfu/g in samples from homes. Although both these sets of samples had been treated before cooking or eating, samples from home treatments were cleaner than those from restaurants [18]. Similar studies showed that the washing of lettuce, cucumber and parsley markedly reduces the content of *L. monocytogenes* [75]. They also studied the influence of the storage temperature, water temperature, acetic acid concentration and immersion time on the survival of *L. monocytogenes*. As expected, the higher storage temperatures increased the number of *L. monocytogenes* colonies. Although washing with dilute acetic acid had some effect on reducing the number of *L. monocytogenes*, the extent of the reduction depended largely on the structure of the vegetable [75]. It is speculated that washing fresh produce to reduce the number of *L. monocytogenes* is more effective in fruits than it is in leafy vegetables.

A survey of *L. monocytogenes* contamination was published on minimally treated leafy vegetables, including collard greens, cabbage, lettuce, Chinese cabbage, and arugula [77]. In total, this research study examined 162 minimally processed leafy samples. Of these, only six samples were confirmed for *Listeria* spp contamination and only three samples were confirmed as *L. monocytogenes,* and these were found in collard greens, bunched parsley and spring onions. Research on market vegetables [78] showed *L. monocytogenes* contamination in 3.1% of the samples. Five salad samples had counts between 1.0 × 10^1^ and 2.6 × 10^2^ cfu/g. Among the minimally processed vegetable samples evaluated in South Korea, 0.3% of them tested positive in sprouts [81]. Uzeh et al. [79] tested many salad vegetables (lettuce, cabbages, carrots and cucumbers), and only cabbages and lettuce showed a positive reaction. Thus, although *L. monocytogenes* levels may decrease after treatment, some colonies could still survive.

## 6. Prevention of Biofilm Formation to Reduce the Level of Contamination

Besides the factors associated with the growing environment, bacterial biofilm formation is an important pathway for fresh produce contamination. Oliveira et al. [82] stated that the term biofilm refers to a sessile form of microbial life, characterized by adhesion of microorganisms to biotic or abiotic surfaces, with consequent production of extracellular polymeric substances.

Fresh produce comes into contact with many different kinds of surfaces at different temperatures during processing or transport, and according to a study by Bonsaglia et al. [83], these two factors influence the extent of *L. monocytogenes* biofilm formation. They compared *L. monocytogenes* biofilms growing on three kinds of touched surfaces, polystyrene, glass and stainless steel, at three different temperatures (4, 20 and 35 °C). The results showed that *L. monocytogenes* attaches more easily to hydrophilic surfaces (glass and stainless steel) than to hydrophobic surfaces (polystyrene). Higher temperatures and longer incubation times decreased the extent of adherence to surfaces, but the results were not significant.

Biofilms are produced by bacteria, including *L. monocytogenes* itself, to enhance their survival and spread. Therefore, disrupting the biofilm of *L. monocytogenes* is a practical method to reduce its survival. Botticella et al. [84] discussed the importance of biofilm formation in relation to the safety of fresh-cut produce. According to them, biofilm formation allows *L. monocytogenes* to persist for long periods of time in the food processing environment and thus represents a source of recurrent contamination and poses a food safety risk. Results reported by Sant’Ana et al. [78] indicated that *L. monocytogenes* persistence either in the field or in the processing environment of the tested RTE vegetables was due to the presence of harborage sites due to biofilm formation. The most common methods employed to reduce biofilm formation include physical (such as UV-C) and chemical (such as chlorine dioxide, peroxyacetic acid) processes.

According to a recent study, physical methods are more effective in controlling biofilm formation because of their minimal influence on product quality and stability [85]. These authors used three physical methods to treat *L. monocytogenes* biofilms: 32 Hz ultra-sonication (US), 390 mJ/cm^2^ Ultraviolet-C (UV-C), and 750 mJ/cm^2^ cold oxygen plasma (COP). UV-C and COP were more effective in reducing *L. monocytogenes* biofilm formation. Another effective method to reduce *L. monocytogenes* biofilm production is to use organic acids combined with modified atmosphere packaging [86]. In that study, by Bae et al. [86], cabbages were treated with 2% lactic acid for 10 min combined with modified atmosphere packaging, and the number of *L. monocytogenes* were reduced by half (from 6.2 cfu/g to 3.1 cfu/g). In addition, the modified atmosphere packaging (air, N_2_ gas, CO_2_ gas) proved to be effective in delaying the growth of *L. monocytogenes*.

## 7. Conclusions

*L. monocytogenes* is widely present in agricultural production environments, and it is implicated in the contamination of fresh crop produce. Most recent listeriosis outbreaks associated with fresh produce are attributed to the crop growing environment, post-harvest processing and retailing. Several reports have demonstrated that *L. monocytogenes* is commonly present in a wide variety of fresh produce samples. It is important to reduce the level of this pathogen to enhance the fresh produce safety and protect consumer health. Preventing *L. monocytogenes* biofilm formation through a practicable and effective method will help to decrease its survival and contamination levels in fresh produce.

## Figures and Tables

**Table 1:** Listeriosis outbreaks associated with fresh produce.

Outbreak Location/Year	Deaths/Cases (% Mortality)	Food Vehicle	References
Boston, USA, 1979	3/20 (15)	Raw vegetables	Ho et al. [56]
Nova Scotia, Canada, 1981	17/41 (41)	Vegetable mix for coleslaw	Schlech et al. [57]
Moncalieri and Giaveno, Italy, 1997	0/2930 (0)	Corn	Aureli et al. [13]
Texas, USA, 2010	5/10 (50)	Chopped celery	Gaul et al. [30]
Colorado, USA, 2011	33/147 (22)	Whole cantaloupes	CDC [54]
Colorado, USA, 2011	15/99 (15)	Lettuce	Shrivastava et al. [55]
Illinois and Michigan, USA, 2014	2/5 (40)	Mung bean sprouts	Garner and Kathariou [58]
California, USA, 2014	1/32 (3)	Caramel apples	CDC [5]
Ohio, USA, 2016	1/19 (5)	Packaged salads	CDC [50]

**Table 2:** Prevalence of *L. monocytogenes* in a fresh produce growing environment.

Country	Environment (Total Number of Samples)	Frequency ^a^ Number of Positive Samples (%)	References
USA	Soil (178)	16 (9%)	Strawn et al. [70]
	Drag swab (175)	15 (9%)	
	Fecal (61)	9 (15%)	
	Water (174)	48 (28%)	
	Engineered (28)	0 (0%)	
	Surface (146)	48 (33%)	
USA	Field	263 (17.5%)	Strawn et al. [71]
	Water	74 (30%)	
Poland	Soil (1000)	55 (5.5%)	Szymczak et al. [72]
Ireland	Soil		McLaughlin et al. [73]
French	soil		Locatelli et al. [74]

^a^ Frequency data represents the number of positive samples (percent of positive samples).

**Table 3:** Some selected studies that reported the prevalence of *L. monocytogenes* in fresh produce.

Produce	Country	Prevalence ^a^	References
Vegetables	China	140 (8, 5.7%)	Wu et al. [76]
Parsley	Poland	30 (3, 10.0%)	Szymczak et al. [72]
	Malaysia	16 (4, 25.0%)	Ponniah et al. [11]
	Brazil	22 (1, 4.5%)	Aparecida de Oliveira et al. [77]
	Greece		Nastou et al. [75]
Collard greens	Brazil	30 (1, 3.3%)	Aparecida de Oliveira et al. [77]
	Brazil	24 (1, 4.2%)	Sant’Ana et al. [78]
Lettuce	Korea		Ding et al. [18]
	Brazil	152 (3, 2.0%)	Sant’Ana et al. [78]
	Nigeria		Uzeh et al. [79]
	Greece		Nastou et al. [75]
Cabbage	Malaysia	32 (7, 21.9%)	Ponniah et al. [11]
	Brazil	11 (2, 18.2%)	Sant’Ana et al. [78]
	Nigeria New Zealand		Uzeh et al. [79] Zhu et al. [80]
Spinach	Brazil	11 (1, 9.1%)	Sant’Ana et al. [78]
Carrot	Malaysia	33 (8, 24.2%)	Ponniah et al. [11]
Tomato	Malaysia	32 (7, 21.9%)	Ponniah et al. [11]
Cucumber	Malaysia	32 (7, 21.9%)	Ponniah et al. [11]
	Greece		Nastou et al. [75]
Sprouts	Korean	112 (1, 0.9%)	Seo et al. [81]

^a^ Number of total analyzed samples (number and percent of positive sample for *L. monocytogenes*).
